# Relative attitude stability analysis of double satellite formation for gravity field exploration in space debris environment

**DOI:** 10.1038/s41598-023-42627-8

**Published:** 2023-09-25

**Authors:** Baocai Pan, Yunhe Meng

**Affiliations:** 1 MOE Key Laboratory of TianQin Mission, TianQin Research Center for Gravitational Physics and School of Physics and Astronomy, Frontiers Science Center for TianQin, CNSA Research Center for Gravitational Waves, Sun Yat-Sen University, Zhuhai, 519082 China; 2 School of Artificial Intelligence, Sun Yat-Sen University, Zhuhai, 519082 China

**Keywords:** Aerospace engineering, Scientific data

## Abstract

Spacecraft operating in low orbit are at risk of being hit by space debris. In the debris environment, the impact of debris is likely to cause the double satellite formation to exit science mode or even lead to the divergence of the control system, thus affecting the scientific exploration mission. In this paper, the attitude stability of the double satellite formation for gravity field in the near circular and polar orbit in the space debris environment is studied. Firstly, based on Lyapunov control and LQR, two sets of control models of stochastic collision for two satellites aligned with each other were proposed, and the actuators were modelled and assigned. Secondly, models of collision probability and momentum are developed. The distribution law of space debris is obtained according to the international common debris software. Meanwhile, probability density function of two independent collisions is gained. Finally, through Monte Carlo simulation and statistics, the changes of relative attitude and thrust torque are simulated when the satellite obtains the angular momentum for a short period of time due to being impacted by space debris. During the 10-year mission period, the number of times that the space debris impact makes the satellite attitude out of the science mode and the number of times that the control system diverges are obtained, which provides a reference for the normal manner of the double satellite formation for gravity field exploration.

## Introduction

In recent years, with the increase of human spaceflight activities, the number of space debris has been increasing. As of 28 July 2021, the number of catalogued debris over 10 cm has reached 23,513, over 1 million space debris between 1 and 10 cm, over 100 million space debris between 1 and 10 mm, and over 20 billion tiny debris below 1 mm are predicted, with masses of thousands of tons^1,2^. If there is no effective way to cope with the situation, in the next 50 years, the number of space debris will increase rapidly at the rate of 10% every year^3^, after 30 years of low Earth orbit no new rail can be used for human^4^, the number of debris after 70 years will reach a limit, this will cause disastrous debris chain collision effect^5,6^.

The large number and wide distribution of tiny debris makes their collision with long-running satellites almost inevitable^1^. When the satellite is impacted by high-speed tiny debris, it will obtain the momentum lost by the debris, and the orbit and attitude will change accordingly^7^. High speed impact of large space debris will have a fatal impact on satellites. More than 16 satellites have been damaged due to debris impact in international public reports and China's satellites have also been invalidated by debris impacts from time to time^4^. In August 2016, Sentinel-1A satellite was hit by a space debris about 1 cm in size, causing the satellite to disintegrate^8^. In March 2022, a piece of space debris that is too small to track collided with the International Space Station, causing damage to a robotic arm. Therefore, it will be a very important research topic to study the collision between space debris and spacecraft under different mission backgrounds. Reference^9^ studied the evolution law of the rotational kinetic energy of space debris when the spacecraft was impacted by space debris at high speed. However, it did not analyze the stability of the spacecraft caused by impact. Since stability is the key to the success of its mission, it is necessary to study the stability maintenance of the spacecraft after being collided under different mission backgrounds.

This paper studies the influence of space debris on the relative attitude stability of the double satellite formation. GRACE—Gravity Recovery and Climate Experiment is the first double satellite formation for gravity field exploration launched by NASA and GFZ. The satellites in the formation are about 220 km apart in near circular and polar orbits. They communicate with each other through microwave, and attitude pointing accuracy is better than 3 mrad. The actuator is composed of magnetorquers and cold air thrusters^10,11^. In 2018, they jointly launched GRACE-FO with the distance between two satellites is 50 km. The change of double satellite distance was measured by laser interferometry, and the attitude pointing accuracy was higher, reaching 0.24 mrad^12^. The satellites for gravity field exploration from single satellite^13,14^ to double satellite formation^10–12^ and from microwave to laser interferometry requires more and more attitude stability, supporting inversion of higher precision on the Earth’s gravity field. Afterwards, ESA and NASA jointly proposed Next Generation Gravity Missions-NGGM with two formations, one with an orbital inclination of 90° and the other of 63°^15^ and China proposed TianQin-2 test satellite.

Since the two satellites of the double satellite formation are identical, the laser beams emitted from the two satellites must be aligned with each other to ensure that the two laser beams can interfere. In other words, the target of the attitude control of the formation is that the outgoing beam of one satellite is aligned with the receiving end of other satellite, and the outgoing beam of the other satellite is aligned with the receiving end of this satellite. If the attitude of one satellite is misaligned, the optical power received on the interferometric signal of other satellite is relatively low, causing inefficient interference efficiency. While the information of angle jitter will be coupled into the distance measurement of the formation, causing measurement bias^16^. Therefore, the maintaining the stability of the relative attitude of the double satellite formation is a key step in ensuring laser interferometric ranging. However, GRACE's satellite development company, Airbus Defense and Space, has only briefly described the control of its attitude^11^, not to mention research on its stability maintenance in the face of complex space environments. Reference^17^ developed two control algorithms based on Lyapunov control and LQR on the condition of attitude control accuracy of GRACE and GRACE-FO. Simulation results show that the controller designed by Lyapunov control algorithm has better comprehensive control effect. However, no relevant research has been found on the impact of space debris on the relative attitude stability of the double satellite formation.

Since the control of two satellites in the double satellite formation for gravity field exploration is almost the same, this paper takes one of the double satellite formation as the research object and establishes a satellite relative attitude dynamics model. The disturbance torque in this model includes the gravity gradient torque and torque caused by the difference of tensor of inertia. The control torques of magnetorquers are described, and the thrust model of cold air thruster is established. The serial link control is adopted for the two actuators. Two control algorithms based on Lyapunov and LQR have been cited in the space debris environment. The number of space debris making satellite attitudes exit science mode that caused by impact, as well as making the control system diverged that obtained under certain control accuracy, and their probability of normal manner has been analysed.

This article first focuses on and analyzes the normal manner of low orbit complex high-precision formation detector systems in debris environments. With the increasing number of space debris, this issue will become more remarkable. Establishing a set of analysis methods and means for the normal manner ability of detector systems has important reference value for the scientific measurement and in orbit operation management of detectors. This paper uses the control algorithm that meets the task requirements to obtain the critical collision of space debris that causes the control system to diverge. A rich supply of data that meets the law of debris distribution is selected, which conducts a Monte Carlo simulation on them. Creatively combining the control algorithm with a Monte Carlo simulation. The control system diverges due to be impacted by space debris in the current task period is simulated through a large amount of data.

The second part is the description of satellite angle motion. The third part is the design of the formation attitude controller and control assignment of the actuator. The fourth part is the probability and momentum modeling of space debris impact, and the fifth part is the simulation and discussion.

## Description of the angular motion

The double satellite formation for the gravity field exploration requires that the two satellites align with each other, and one satellite always points to the other accurately. The double satellite formation is composed of two satellites with almost identical motion and control. Therefore, this paper takes one of the satellites as the research object and controls its body frame to coincide with the reference frame within the error range. To simplify the study, we assume here that the reference frame is known and not affected by space debris impacts. The definition of coordinate frame is as follows.

- $O_{I} - X_{I} Y_{I} Z_{I}$ inertial frame—IF. The origin is located in the Earth center of mass, $O_{I} Z_{I}$ is the rotation axis of the Earth and $O_{I} X_{I}$ points to the vernal equinox of J2000 epoch.
- $o_{O} - x_{O} y_{O} z_{O}$ orbit frame—OF. The origin is located in the satellite center of mass, $o_{O} z_{O}$ points to the Earth center, and $o_{O} x_{O}$ is located in the orbit plane perpendicular to $o_{O} z_{O}$ and pointing to the direction of motion.
- $o_{B} - x_{B} y_{B} z_{B}$ body frame—BF. Its axes are the principal axes of inertia for the satellite.$o_{B} x_{B}$ is the sight line of the laser, and $o_{B} z_{B}$ is perpendicular to the bottom of the satellite.
- $o_{R} - x_{R} y_{R} z_{R}$ reference frame—RF. The origin is the midpoint of the line from following satellite to the main satellite, where $o_{R} x_{R}$ points from the follow satellite to the main satellite, and $o_{R} z_{R}$ is perpendicular to $o_{R} x_{R}$ in the orbit plane.

**Figure 1:**
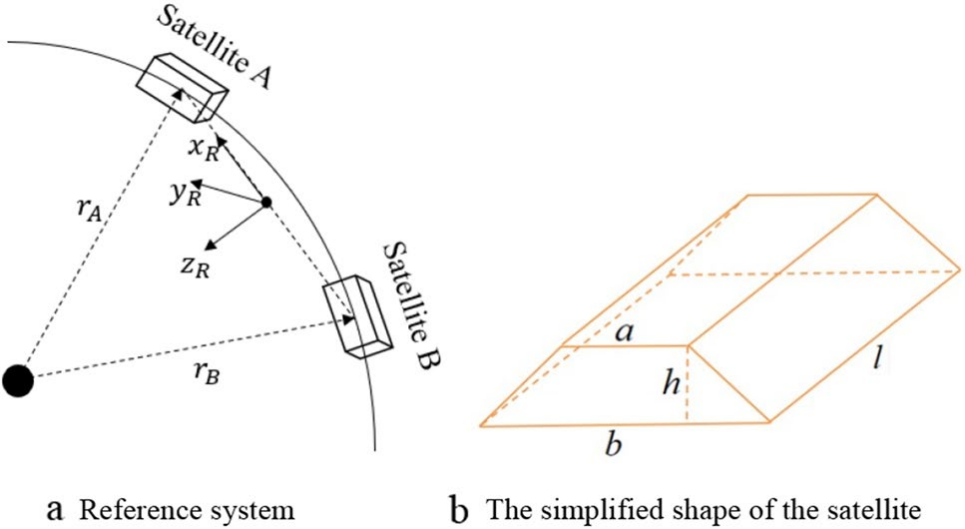
Double-satellite formation and satellite shape.

The reference frame of the double satellite formation is shown in Fig. 1a, and the simplified shape of the satellite is shown in Fig. 1b

${\mathbf{i}}_{1} ,{\mathbf{i}}_{2} ,{\mathbf{i}}_{3}$ and ${\mathbf{e}}_{1} ,{\mathbf{e}}_{2} ,{\mathbf{e}}_{3}$ are the unit vectors of the $x,y,z$ axis under the orbital and reference frames, respectively, and the unit vector is defined as follows

$${\mathbf{i}}_{3} = - \frac{{{\mathbf{r}}_{B} }}{{\left| {{\mathbf{r}}_{B} } \right|}},{\mathbf{i}}_{2} = \frac{{{\mathbf{v}}_{B} \times {\mathbf{r}}_{B} }}{{\left| {{\mathbf{v}}_{B} \times {\mathbf{r}}_{B} } \right|}},{\mathbf{i}}_{1} = {\mathbf{i}}_{2} \times {\mathbf{i}}_{3}$$

$$\begin{gathered} {\mathbf{e}}_{1} {\mathbf{ = }}{{\left[ {{{\left( {{\mathbf{r}}_{{\mathbf{A}}} {\mathbf{ - r}}_{{\mathbf{B}}} } \right)} \mathord{\left/ {\vphantom {{\left( {{\mathbf{r}}_{{\mathbf{A}}} {\mathbf{ - r}}_{{\mathbf{B}}} } \right)} 2}} \right. \kern-0pt} 2}} \right]} \mathord{\left/ {\vphantom {{\left[ {{{\left( {{\mathbf{r}}_{{\mathbf{A}}} {\mathbf{ - r}}_{{\mathbf{B}}} } \right)} \mathord{\left/ {\vphantom {{\left( {{\mathbf{r}}_{{\mathbf{A}}} {\mathbf{ - r}}_{{\mathbf{B}}} } \right)} 2}} \right. \kern-0pt} 2}} \right]} {\left| {\left[ {{{\left( {{\mathbf{r}}_{{\mathbf{A}}} {\mathbf{ - r}}_{{\mathbf{B}}} } \right)} \mathord{\left/ {\vphantom {{\left( {{\mathbf{r}}_{{\mathbf{A}}} {\mathbf{ - r}}_{{\mathbf{B}}} } \right)} 2}} \right. \kern-0pt} 2}} \right]} \right|}}} \right. \kern-0pt} {\left| {\left[ {{{\left( {{\mathbf{r}}_{{\mathbf{A}}} {\mathbf{ - r}}_{{\mathbf{B}}} } \right)} \mathord{\left/ {\vphantom {{\left( {{\mathbf{r}}_{{\mathbf{A}}} {\mathbf{ - r}}_{{\mathbf{B}}} } \right)} 2}} \right. \kern-0pt} 2}} \right]} \right|}} \hfill \\ {\mathbf{e}}_{2} = {{\left[ {{{{\mathbf{e}}_{1} \times \left( {{\mathbf{r}}_{{\mathbf{A}}} + {\mathbf{r}}_{{\mathbf{B}}} } \right)} \mathord{\left/ {\vphantom {{{\mathbf{e}}_{1} \times \left( {{\mathbf{r}}_{{\mathbf{A}}} + {\mathbf{r}}_{{\mathbf{B}}} } \right)} 2}} \right. \kern-0pt} 2}} \right]} \mathord{\left/ {\vphantom {{\left[ {{{{\mathbf{e}}_{1} \times \left( {{\mathbf{r}}_{{\mathbf{A}}} + {\mathbf{r}}_{{\mathbf{B}}} } \right)} \mathord{\left/ {\vphantom {{{\mathbf{e}}_{1} \times \left( {{\mathbf{r}}_{{\mathbf{A}}} + {\mathbf{r}}_{{\mathbf{B}}} } \right)} 2}} \right. \kern-0pt} 2}} \right]} {\left| {\left[ {{{{\mathbf{e}}_{1} \times \left( {{\mathbf{r}}_{{\mathbf{A}}} + {\mathbf{r}}_{{\mathbf{B}}} } \right)} \mathord{\left/ {\vphantom {{{\mathbf{e}}_{1} \times \left( {{\mathbf{r}}_{{\mathbf{A}}} + {\mathbf{r}}_{{\mathbf{B}}} } \right)} 2}} \right. \kern-0pt} 2}} \right]} \right|}}} \right. \kern-0pt} {\left| {\left[ {{{{\mathbf{e}}_{1} \times \left( {{\mathbf{r}}_{{\mathbf{A}}} + {\mathbf{r}}_{{\mathbf{B}}} } \right)} \mathord{\left/ {\vphantom {{{\mathbf{e}}_{1} \times \left( {{\mathbf{r}}_{{\mathbf{A}}} + {\mathbf{r}}_{{\mathbf{B}}} } \right)} 2}} \right. \kern-0pt} 2}} \right]} \right|}} \hfill \\ {\mathbf{e}}_{3} = {\mathbf{e}}_{1} \times {\mathbf{e}}_{2} \hfill \\ \end{gathered}$$

The rotation matrix and its derivatives from the inertial frame to the reference system are

$${\mathbf{R}}_{ref} = \left[ {\begin{array}{*{20}l} {{\mathbf{e}}_{1} } & {{\mathbf{e}}_{2} } & {{\mathbf{e}}_{3} } \\ \end{array} } \right]^{T} ,{\dot{\mathbf{R}}}_{ref} = \left[ {\begin{array}{*{20}l} {{\dot{\mathbf{e}}}_{1} } & {{\dot{\mathbf{e}}}_{2} } & {{\dot{\mathbf{e}}}_{3} } \\ \end{array} } \right]^{T} .$$

The reference angular velocity expressed without collision with space in the body frame is

$$\left[ {{{\varvec{\upomega}}}_{ref} } \right]_{ \times } = - {\dot{\mathbf{R}}}_{ref} {\mathbf{R}}_{ref}^{T} .$$

The skew symmetric matrix of cross product is

$$\left[ {{\varvec{\upomega}}} \right]_{ \times } = \left[ {\begin{array}{*{20}l} 0 & { - \omega_{3} } & {\omega_{2} } \\ {\omega_{3} } & 0 & { - \omega_{1} } \\ { - \omega_{2} } & {\omega_{1} } & 0 \\ \end{array} } \right].$$

## Attitude control of the double satellite formation

### Lyapunov attitude control

The attitude dynamics equation of the satellite moving around the center of mass is

1 $$\left\{ {\begin{array}{*{20}l} {{\mathbf{J}\dot{\varvec{\mathbf{\omega }}}}_{{{\text{abs}}}} + {{\varvec{\upomega}}}_{{{\text{abs}}}} \times {\mathbf{J\omega }}_{abs} = {\mathbf{M}}_{ext} + {\mathbf{M}}_{ctrl} + {\mathbf{M}}_{impact} } \\ {{\dot{\mathbf{Q}}} = - \left[ {{{\varvec{\upomega}}}_{abs} } \right]_{ \times } {\mathbf{Q}}} \\ \end{array} } \right.$$

where,${\mathbf{J}} = diag\left( {J_{1} ,J_{2} ,J_{3} } \right)$,${{\varvec{\upomega}}}_{abs}$ is angular velocity of satellite relative inertial frame, ${\mathbf{M}}_{ctrl} \in {\mathbb{R}}^{n \times 1}$ is the control torque applied to the satellite, ${\mathbf{M}}_{ext} \in {\mathbb{R}}^{n \times 1}$ is the external disturbance torque, and ${\mathbf{M}}_{impact} \in {\mathbb{R}}^{n \times 1}$ is the instantaneous torque obtained after the satellite is impacted. ${\mathbf{Q}}$ is the rotation matrix from the inertial frame to body frame expressed in terms of Euler angle. The relative angular velocity and angular acceleration from body frame to the reference frame are ${{\varvec{\upomega}}} = {{\varvec{\upomega}}}_{abs} - {\mathbf{D{\varvec{\omega}} }}_{ref}$ and ${\dot{\varvec{\omega }}} = {\dot{\varvec{\omega }}}_{abs} - {\mathbf{D}\dot{{\varvec{\mathbf{\omega}} }}}_{ref} + {{\varvec{\upomega}}} \times {\mathbf{D}{\varvec{\mathbf{\omega}} }}_{ref}$ respectively, where ${\mathbf{D}}$ is the rotation matrix from the reference frame to the body frame.

Equations (2–5) are obtained from references^17,18^.

2 $$V = \frac{1}{2}\left( {\omega ,{\mathbf{J}}\omega } \right) + k_{a} \left( {3 - tr{\mathbf{D}}} \right),k_{a} = const > 0$$

The derivative of this function with respect to time is

3 $$\dot{V} = \left( {\omega ,{\mathbf{J}}\dot{\omega }} \right) - k_{a} tr{\dot{\mathbf{D}}} = \left( {\omega ,{\mathbf{J}}\dot{\omega }_{abs} + {\mathbf{J}}\left[ \omega \right]_{ \times } {\mathbf{D}}\omega_{ref} - {\mathbf{JD}}\dot{\omega }_{ref} + k_{a} {\mathbf{S}}} \right)$$

where, ${\mathbf{S}} = \left( {D_{23} - D_{32} ,D_{31} - D_{13} ,D_{12} - D_{21} } \right)$,$D_{ij}$ is the element corresponding to the rotation matrix ${\mathbf{D}}$.

If the expression satisfies the following equation

4 $${\mathbf{J}}\dot{\omega }_{abs} + {\mathbf{J}}\left[ \omega \right]_{ \times } {\mathbf{D}}\omega_{ref} - {\mathbf{JD}}\dot{\omega }_{ref} + k_{a} {\mathbf{S}} = - k_{\omega } \omega ,k_{\omega } = const > 0.$$

The control torque is

5 $${\mathbf{M}}_{ctrl} = \omega_{abs} \times {\mathbf{J}}\omega_{abs} - {\mathbf{M}}_{ext} + {\mathbf{M}}_{impact} - {\mathbf{J}}\left[ \omega \right]_{ \times } {\mathbf{D}}\omega_{ref} + {\mathbf{JD}}\dot{\omega }_{ref} - k_{\alpha } {\mathbf{S}} - k_{\omega } \omega .$$

### Dynamic equation in vicinity of equilibrium

It is difficult to accurately calculate all the external interference torque ${\mathbf{M}}_{ext}$ for the satellite, so the external torque in this paper includes gravity gradient torque, and torque caused by the change of tensor of inertia, which are respectively

6 $$\left\{ {\begin{array}{*{20}l} {{\mathbf{M}}_{grav} = 3\frac{\mu }{{r^{5} }}{\mathbf{r}} \times {\mathbf{Jr}}} \\ {{\mathbf{M}}_{in} = \left( {\omega + {\mathbf{D}}\omega_{ref} } \right) \times \delta {\mathbf{J}}\left( {\omega + {\mathbf{D}}\omega_{ref} } \right) + \delta {\mathbf{J}}\left( { - \left[ \omega \right]_{ \times } {\mathbf{D}}\omega_{ref} + {\mathbf{D}}\dot{\omega }_{ref} } \right)} \\ \end{array} } \right.$$

The rotation matrix ${\mathbf{D}}$ rotates in the order of 2-3-1. $\alpha_{1} ,\alpha_{2}$ and $\alpha_{3}$ represent roll, pitch and yaw respectively.

$${\mathbf{D}}_{312} = \left[ {\begin{array}{*{20}l} {\cos \alpha_{2} \cos \alpha_{3} } & {\sin \alpha_{3} } & { - \sin \alpha_{2} \cos \alpha_{3} } \\ { - \cos \alpha_{1} \cos \alpha_{2} \sin \alpha_{3} + \sin \alpha_{1} \sin \alpha_{2} } & {\cos \alpha_{1} \cos \alpha_{3} } & {\cos \alpha_{1} \sin \alpha_{2} \sin \alpha_{3} + \sin \alpha_{1} \cos \alpha_{2} } \\ {\sin \alpha_{1} \cos \alpha_{2} \sin \alpha_{3} + \cos \alpha_{1} \sin \alpha_{2} } & { - \sin \alpha_{1} \cos \alpha_{3} } & { - \sin \alpha_{1} \sin \alpha_{2} \sin \alpha_{3} + \cos \alpha_{1} \cos \alpha_{2} } \\ \end{array} } \right]$$

Linearized in vicinity of equilibrium, the expression omits that the second order minima

$${\mathbf{D}} \approx {\mathbf{I}}_{3} - \left[ \alpha \right]_{ \times } ,{\text{then}}\,{\dot{\mathbf{D}}} \approx - \left[ \omega \right]_{ \times } ,{\mathbf{S}} = 2\left( {\alpha_{1} ,\alpha_{2} ,\alpha_{3} } \right)^{T} = 2{{\varvec{\upalpha}}},\sin \alpha \approx \alpha ,\cos \alpha \approx 1.$$

To sum up, the relative angular motion equation of the satellite omitting high-order small quantities is

7 $$\left\{ {\begin{array}{*{20}l} {{\dot{\varvec{\alpha }}} = {{\varvec{\upomega}}}\quad \quad \quad \quad \quad \quad \quad \quad \quad \quad \quad \quad \quad \quad \quad \;\;\;} \\ {{\dot{\varvec{\omega }}} = {\mathbf{A}}_{1} {{\varvec{\upomega}}} + {\mathbf{A}}_{2} {{\varvec{\upalpha}}} + {\mathbf{J}}^{ - 1} \left\{ {\left[ {{{\varvec{\upomega}}}_{ref} } \right]_{ \times } \delta {\mathbf{J{\varvec{\omega}} }}_{ref} + {\mathbf{M}}_{impact} } \right\},} \\ \end{array} } \right.$$

where

$${\mathbf{A}}_{{\mathbf{1}}} = {\mathbf{J}}^{ - 1} \left\{ { - {\mathbf{J}}\left[ {{{\varvec{\upomega}}}_{ref} } \right]_{ \times } + \left[ {{\mathbf{J}}\omega_{ref} } \right]_{ \times } - \left[ {{{\varvec{\upomega}}}_{ref} } \right]{\mathbf{J}} - \left[ {\delta {\mathbf{J\omega }}_{ref} } \right]_{ \times } + \left[ {{{\varvec{\upomega}}}_{ref} } \right]_{ \times } \delta {\mathbf{J}} + \delta {\mathbf{J}}\left[ {{{\varvec{\upomega}}}_{ref} } \right]_{ \times } + k_{\omega } } \right\},$$

$$\begin{aligned} {\mathbf{A}}_{{\mathbf{2}}} & = {\mathbf{J}}^{ - 1} \left\{ { - \left[ {{{\varvec{\upomega}}}_{ref} } \right]_{ \times } {\mathbf{J}}\left[ {{{\varvec{\upomega}}}_{ref} } \right]_{ \times } - 3\omega_{0}^{2} \left( { - \left[ {{\mathbf{e}}_{1} } \right]_{ \times } {\mathbf{J}}\left[ {{\mathbf{e}}_{1} } \right]_{ \times } + \left[ {{\mathbf{Je}}_{1} } \right]_{ \times } \left[ {{\mathbf{e}}_{1} } \right]_{ \times } } \right)} \right. \\ & \quad \left. { + \left[ {{\mathbf{J}}\omega_{ref} } \right]_{ \times } \left[ {{{\varvec{\upomega}}}_{ref} } \right]_{ \times } - \left[ {{{\varvec{\upomega}}}_{ref} } \right]_{ \times } \delta {\mathbf{J}}\left[ {{{\varvec{\upomega}}}_{ref} } \right]_{ \times } \left[ {\delta {\mathbf{J{\varvec{\omega}} }}_{ref} } \right]_{ \times } \left[ {{{\varvec{\upomega}}}_{ref} } \right]_{ \times } + 2k_{\alpha } } \right\}, \\ \end{aligned}$$

$${{\varvec{\upomega}}} = {\dot{\varvec{\alpha }}},{\mathbf{e}}_{1} = {{\mathbf{r}} \mathord{\left/ {\vphantom {{\mathbf{r}} r}} \right. \kern-0pt} r} = \left( {0,0, - 1} \right)^{T} ,{{\varvec{\upalpha}}} = \left( {\alpha_{1} ,\alpha_{2} ,\alpha_{3} } \right)^{T} .$$

### Control torques generated by magnetorquers

The magnetorquers are installed along the body frame of the satellite. Three magnetic torques in mutually perpendicular directions are generated by the action of the external magnetic field. However, the control accuracy of the magnetorquers can only reach the order of degrees, which is far from meeting the requirements of attitude control accuracy. It also needs to be combined with other actuators to meet the requirements of the mission^19,20^.

The control torque generated by the magnetorquers is

8 $${\mathbf{M}}_{ctrl1} = {\mathbf{m}} \times {\mathbf{B}},$$

where ${\mathbf{m}}$ is the magnetic dipole vector and ${\mathbf{B}}$ is the magnetic induction intensity of the Earth. According to the control torque equation, the control torque is always orthogonal to the direction of magnetic induction intensity. When the satellite is near the equator, the magnetic torque can only control the pitch and yaw angle. When the satellite is near the poles, the magnetic torque can only control the roll and pitch angle. This means that at any time, there is always a direction where the magnetorquers cannot generate control torque.

The dipole moment is generated by the electromagnetic coil in the magnetorquers, which is composed of electromagnetic materials with high permeability. Here, we assume that the input current does not exceed $\pm 110\,{\text{mA}}$ and the maximum magnetic dipole moment does not exceed $\pm 27.5\,{\text{A}}\,{\text{m}}^{2}$^21^.

The magnetic induction intensity of the Earth is ${\mathbf{B}}$, which is expressed in the orbital frame^22, 23^

9 $${\mathbf{B}}^{OF} = \frac{{\mu_{B} }}{{r^{3} }}\left[ {\begin{array}{*{20}l} {\sin i\cos u} \\ { - \cos i} \\ {2\sin i\sin u} \\ \end{array} } \right],$$

where $\mu_{B}$ is the geomagnetic constant, $i$ is the orbital inclination, $u$ is the latitude argument and its expression is $u = \omega_{0} t + u_{0}$, $\omega_{0}$ is the angular velocity of the satellite.

The control torque generated by the magnetorquer is

10 $${\mathbf{M}}_{ctrl1} = - \left[ {{\mathbf{e}}_{B} } \right]_{ \times } \left[ {{\mathbf{e}}_{B} } \right]_{ \times } \left( { - {\mathbf{JA}}_{2} {{\varvec{\upalpha}}} - {\mathbf{JA}}_{1} {{\varvec{\upomega}}} - k_{\alpha } {{\varvec{\upalpha}}} - k_{\omega } {{\varvec{\upomega}}}} \right),$$

where, $${\mathbf{e}}_{B} = \frac{{\mathbf{B}}}{{\sqrt {\left( {{\mathbf{B}},{\mathbf{B}}} \right)} }}$$.

### Control algorithm of LQR

For optimal control of linear quadratic regulator (LQR), the cost function and system state equation are as follows

11 $$\left\{ {\begin{array}{*{20}l} {{\dot{\mathbf{x}}} = {\mathbf{A}}\left( t \right){\mathbf{x}} + {\mathbf{B}}_{ctrl} \left( t \right){\mathbf{u}} + {\mathbf{B}}_{d} {\mathbf{w}}\quad \quad \quad \;\;\;\;} \\ {J = {\mathbf{x}}_{f}^{T} {\mathbf{Px}}_{f} + \int_{0}^{{T_{f} }} {{\mathbf{x}}^{T} {\mathbf{Q}}\left( t \right)} {\mathbf{x}} + {\mathbf{u}}^{T} {\mathbf{R}}\left( t \right){\mathbf{u}}^{T} dt,} \\ \end{array} } \right.$$

where,

$${\mathbf{A}}\left( t \right) = \left[ {\begin{array}{*{20}l} {0_{3} } & {{\mathbf{I}}_{3} } \\ {{\mathbf{A}}_{lqr1} } & {{\mathbf{A}}_{lqr2} } \\ \end{array} } \right],$$

$${\mathbf{B}}_{ctrl} \left( t \right) = \left[ {\begin{array}{*{20}l} {0_{3} } & {0_{3} } \\ { - {\mathbf{J}}^{ - 1} \left[ {\mathbf{B}} \right]_{ \times } } & { - {\mathbf{J}}^{ - 1} diag\left( {B_{1} ,B_{2} ,B_{3} } \right)} \\ \end{array} } \right],$$

$${\mathbf{A}}_{lqr1} = {\mathbf{J}}^{ - 1} \left\{ { - {\mathbf{J}}\left[ {{{\varvec{\upomega}}}_{ref} } \right]_{ \times } + \left[ {{\mathbf{J{\varvec{\omega}} }}_{ref} } \right]_{ \times } - \left[ {{{\varvec{\upomega}}}_{ref} } \right]_{ \times } {\mathbf{J}} - \left[ {\delta {\mathbf{J{\varvec{\omega}} }}_{ref} } \right]_{ \times } + \left[ {{{\varvec{\upomega}}}_{ref} } \right]_{ \times } \delta {\mathbf{J}} + \delta {\mathbf{J}}\left[ {{{\varvec{\upomega}}}_{ref} } \right]_{ \times } } \right\},$$

$$\begin{aligned} {\mathbf{A}}_{lqr2} & = {\mathbf{J}}^{ - 1} \left\{ { - \left[ {{{\varvec{\upomega}}}_{ref} } \right]_{ \times } {\mathbf{J}}\left[ {{{\varvec{\upomega}}}_{ref} } \right]_{ \times } - 3\omega_{0}^{2} \left( { - \left[ {{\mathbf{e}}_{1} } \right]_{ \times } {\mathbf{J}}\left[ {{\mathbf{e}}_{1} } \right]_{ \times } + \left[ {{\mathbf{Je}}_{1} } \right]_{ \times } \left[ {{\mathbf{e}}_{1} } \right]_{ \times } } \right)} \right. \\ & \quad \left. { + \left[ {{\mathbf{J{\varvec{\omega}} }}_{ref} } \right]_{ \times } \left[ {{{\varvec{\upomega}}}_{ref} } \right]_{ \times } + \left[ {{{\varvec{\upomega}}}_{ref} } \right]_{ \times } \delta {\mathbf{J}}\left[ {{{\varvec{\upomega}}}_{ref} } \right]_{ \times } - \left[ {\delta {\mathbf{J{\varvec{\omega }}}}_{ref} } \right]_{ \times } \left[ {{{\varvec{\upomega}}}_{ref} } \right]_{ \times } } \right\}, \\ \end{aligned}$$

$${\mathbf{w}} = {\mathbf{J}}^{ - 1} \left\{ {\left[ {{{\varvec{\upomega}}}_{ref} } \right]_{ \times } \delta {\mathbf{J{\varvec{\omega}} }}_{ref} + {\mathbf{M}}_{impact} } \right\}.$$

${\mathbf{x}}$ is the state vector, $T_{f}$ is the final time, ${\mathbf{P}}$ is a positive definite symmetric constant matrix, ${\mathbf{Q}}$ and ${\mathbf{R}}$ are positive definite symmetric time-varying matrices, and ${\mathbf{u}}$ is the control torque. ${\mathbf{A}}$ is the dynamics matrix, ${\mathbf{B}}_{ctrl}$ is the control matrix, ${\mathbf{w}}$ is the model noise, ${\mathbf{B}}_{d}$ is the noise coefficient, and ${\mathbf{B}}$ is the magnetic induction intensity.

The optimal control is

12 $${\mathbf{u}} = - {\mathbf{R}}^{ - 1} \left( t \right){\mathbf{B}}_{ctrl}^{T} \left( t \right){\mathbf{P}}\left( t \right){\mathbf{x}}\left( t \right).$$

The magnetic dipole vector matrix is ${\mathbf{m}} = {\mathbf{u}}$. The differential Riccati equation of ${\mathbf{P}}\left( t \right)$ is

13 $${\dot{\mathbf{P}}}\left( t \right) + {\mathbf{P}}\left( t \right){\mathbf{A}}\left( t \right) + {\mathbf{A}}^{T} \left( t \right){\mathbf{P}}\left( t \right) - {\mathbf{P}}\left( t \right){\mathbf{B}}_{ctrl} \left( t \right){\mathbf{R}}^{ - 1} \left( t \right){\mathbf{B}}_{ctrl}^{T} \left( t \right){\mathbf{P}}\left( t \right) + {\mathbf{Q}}\left( t \right) = 0.$$

The boundary condition of the equation is ${\mathbf{P}}\left( {T_{f} } \right) = {\mathbf{P}}_{f}$.

### Control distribution of actuator

**Figure 2:**
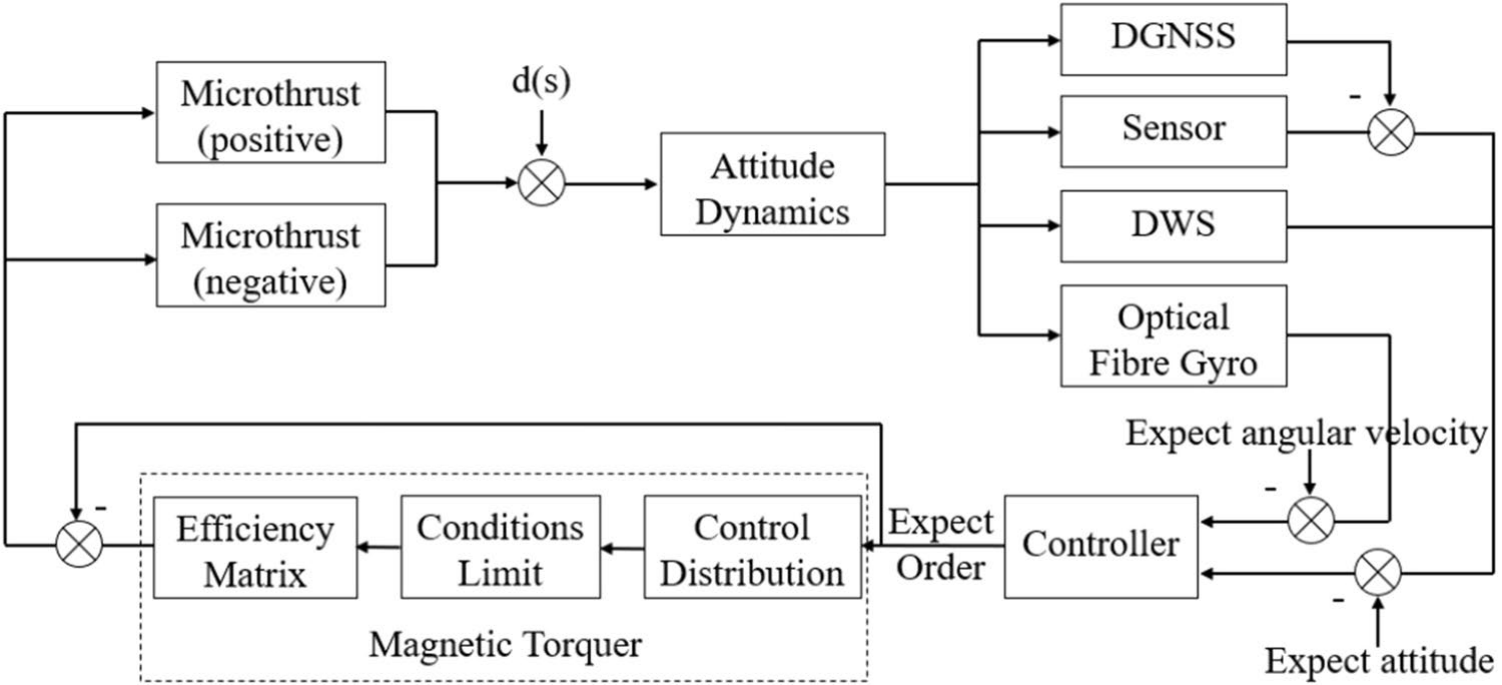
Attitude control system based on serial link allocation.

For a system composed of two or more actuators, how to reasonably assign virtual expected instructions to each actuator to satisfy the stability requirements of spacecraft attitude is the problem of actuator control allocation. The common method is to incorporate the control allocation of the actuator into the design of the control law. Considering the applicability of the project, this paper adopts a simple and practical string link allocation rule. This distribution rule assumes that different actuators provide control torque according to different priorities. After the actuators with higher priorities reach saturation, the remaining execution instructions are completed by the next level of actuators. As shown in Fig. 2, the control command of virtual expectation can be converted into the control command of each actuator only after control allocation.

The control allocation expression form is as follows

14 $${\mathbf{B}}_{eff} {\mathbf{u}}\left( t \right) = {\mathbf{v}}\left( t \right),$$

where, ${\mathbf{v}}\left( t \right)$ is the desired virtual control instruction, ${\mathbf{u}}\left( t \right)$ is the input instruction of the actuator, and ${\mathbf{B}}_{eff}$ is the control efficiency matrix. The control allocation problem can be transformed into

15 $$B_{1} u_{1} + B_{2} u_{2} = v.$$

The actuator uses a magnetic torquer for control distribution first, that is, $B_{1} u_{1} = v_{1}$. We know that it is difficult to meet the control requirements only with a magnetic torquer, that is $v_{1} \le v$, therefore, $v_{1\max } = B_{1} u_{1}$, $v_{2} = v - B_{1} u_{1}$.

In order to control a certain attitude of the satellite, two heterogeneous actuators need to cooperate with each other to complete the control task, that is, the input torque $u\left( t \right)$ of the actuator is not unique. Since the magnetic dipole vector is limited to 27.5 ${\text{A}}\,{\text{m}}^{2}$ and the maximum thrust of the thruster is 10 ${\text{mN}}$, the actual performance index of the actuator is limited by physical conditions, and the input variable $u\left( t \right)$ satisfies the inequality $u_{\min } \le {\mathbf{u}}\left( t \right) \le u_{\max }$. Different actuators have different corresponding rates of instructions. This characteristic is represented by the rate $\dot{u}\left( t \right)$ of output instruction $u\left( t \right)$, i.e. $\rho_{\min } \le \dot{u}\left( t \right) \le \rho_{\min }$. Therefore, the restrictions of actuators are

16 $$\left\{ {\begin{array}{*{20}l} {u_{\min } \le {\mathbf{u}}\left( t \right) \le u_{\max } } \\ {\rho_{\min } \le {\dot{\mathbf{u}}}\left( t \right) \le \rho_{\max } } \\ {{\dot{\mathbf{u}}}\left( t \right) = {{\left[ {{\mathbf{u}}\left( t \right) - {\mathbf{u}}\left( {t - 1} \right)} \right]} \mathord{\left/ {\vphantom {{\left[ {{\mathbf{u}}\left( t \right) - {\mathbf{u}}\left( {t - 1} \right)} \right]} T}} \right. \kern-0pt} T}.} \\ \end{array} } \right.$$

### Control torque generated by thruster

It is difficult to achieve the attitude control accuracy by using only the torque generated by the magnetorquers. Therefore, micro thrusters with a maximum thrust of 10 ${\text{mN}}$ need to be installed on the satellite, in order to achieve the attitude control accuracy of the satellite. Because it will produce a large torque in a short time, the attitude and angular velocity of the satellite will change almost simultaneously when the micro thruster is working. When the relative attitude and relative angular velocity reach the allowable boundary, the actuator starts to work. It should be noted that the switch of thruster does not consider the delay phenomenon. The control torque ${\mathbf{M}}_{ctrl2}$ generated by the micro thruster is

17 $${\mathbf{M}}_{ctrl2i} \left( {\alpha ,\dot{\alpha }} \right) = \left\{ {\begin{array}{*{20}l} { - {\mathbf{B}}_{ctrl2} {\mathbf{u}}_{ctrl2i} ,if\alpha > \alpha_{0} \;or\;\dot{\alpha } > - \dot{\alpha }_{0} } \\ {{\mathbf{B}}_{ctrl2} {\mathbf{u}}_{ctrl2i} ,if\alpha < - \alpha_{0} \;or\;\dot{\alpha } < \dot{\alpha }_{0} } \\ {0,otherwise,} \\ \end{array} } \right.$$

18 $${\mathbf{B}}_{ctrl2} = \left[ {\begin{array}{*{20}l} {0_{3} } \\ {diag\left( {B_{1} ,B_{2} ,B_{3} } \right)} \\ \end{array} } \right],$$

19 $${\mathbf{u}}_{ctrl2i} = \left\{ {\begin{array}{*{20}l} { - \left[ {\begin{array}{*{20}l} {diag\left( {{\mathbf{k}}_{\omega } } \right) + {\mathbf{A}}_{\omega } } & {0_{3 \times 3} } \\ {0_{3 \times 3} } & {diag\left( {{\mathbf{k}}_{\alpha } } \right) + {\mathbf{A}}_{\alpha } } \\ \end{array} } \right] \cdot \left( {\left[ {\begin{array}{*{20}l} {{\dot{\mathbf{\alpha }}}_{i} } \\ {{{\varvec{\upalpha}}}_{i} } \\ \end{array} } \right] + \left[ {\begin{array}{*{20}l} {\delta {\dot{\mathbf{\alpha }}}_{i} } \\ {\delta {{\varvec{\upalpha}}}_{i} } \\ \end{array} } \right]} \right),Lyapunov} \\ {\,\quad - {\mathbf{R}}^{ - 1} {\mathbf{B}}_{ctrli}^{T} {\mathbf{P}} \cdot \left( {\left[ {\begin{array}{*{20}l} {{\dot{\varvec{\alpha }}}_{i} } \\ {{{\varvec{\upalpha}}}_{i} } \\ \end{array} } \right] + \left[ {\begin{array}{*{20}l} {\delta {\dot{\varvec{\alpha }}}_{i} } \\ {\delta {{\varvec{\upalpha}}}_{i} } \\ \end{array} } \right]} \right),LQR\quad \quad \quad \quad \quad \quad \quad \quad \quad \quad \quad \quad \quad } \\ \end{array} } \right.,$$

where

$${\mathbf{A}}_{\alpha } = \left[ {\begin{array}{*{20}l} {4\omega_{0}^{2} \left( {J_{3} - J_{2} } \right)} & 0 & 0 \\ 0 & {3\omega_{0}^{2} \left( {J_{3} - J_{1} } \right)} & 0 \\ 0 & 0 & {\omega_{0}^{2} \left( {J_{1} - J_{2} } \right)} \\ \end{array} } \right],$$

$${\mathbf{A}}_{\omega } = \left[ {\begin{array}{*{20}l} 0 & 0 & {\omega_{0} \left( {J_{3} + J_{1} - J_{2} } \right)} \\ 0 & 0 & 0 \\ {\omega_{0} \left( {J_{2} - J_{3} - J_{1} } \right)} & 0 & 0 \\ \end{array} } \right].$$

In order to solve the approximate values of ${\mathbf{k}}_{\alpha } ,{\mathbf{k}}_{\omega }$, using Floquet theory, the approximate initial condition of system (7) is $\Phi \left( 0 \right) = I_{6 \times 6}$, and $\rho_{k}$ is the characteristic root of characteristic equation $\det \left( {\Phi \left( T \right) - \rho_{k} I_{6 \times 6} } \right) = 0$. To make the linear system $\left( {\alpha ,\omega } \right)^{T} = 0$ asymptotically stable in a large range near the equilibrium position, the inequality $k{\text{Re}} \left( {\ln \rho_{k} } \right) < 0$ for any $\rho_{k}$ is constant. When $\max \left[ {{\text{Re}} \left( {\ln \rho_{k} } \right)} \right]$ is the smallest, the system returns to the equilibrium position faster. Therefore, the most suitable control parameters for system stability are obtained through $\mathop {\max }\limits_{k} \left[ {{\text{Re}} \left( {\ln \rho_{k} } \right)} \right] \to \min$.

## Probability and momentum of collision

In this paper, the average orbital height of the formation is $h = 450km$, eccentricity is $e = 0.001$, orbit inclination is $i = 89.5^{ \circ }$, and average effective cross-sectional area is $S \approx 3m^{2}$. Space debris was assessed using the international common software ORDEM2000. From the software ORDEM2000, the flux of space debris with a size greater than or equal to 0.1 mm over a 10-year mission period is $\Phi = {{1427.5639} \mathord{\left/ {\vphantom {{1427.5639} {3\,}}} \right. \kern-0pt} {3\,}}{{{\text{m}}^{2} } \mathord{\left/ {\vphantom {{{\text{m}}^{2} } {10\,{\text{year}}}}} \right. \kern-0pt} {10\,{\text{year}}}}$, the flux of debris with a size greater than or equal to 1 mm is $\Phi = {{3.0216} \mathord{\left/ {\vphantom {{3.0216} 3}} \right. \kern-0pt} 3}{{\,{\text{m}}^{2} } \mathord{\left/ {\vphantom {{\,{\text{m}}^{2} } {10\,{\text{year}}}}} \right. \kern-0pt} {10\,{\text{year}}}}$, and the flux of debris impacted with a size greater than or equal to 10 mm is $\Phi = {{3.0734 \times 10^{ - 4} } \mathord{\left/ {\vphantom {{3.0734 \times 10^{ - 4} } 3}} \right. \kern-0pt} 3}\,{{{\text{m}}^{2} } \mathord{\left/ {\vphantom {{{\text{m}}^{2} } {10\,{\text{year}}}}} \right. \kern-0pt} {10\,{\text{year}}}}$。Therefore, the probability of the formation being impacted by space debris of centimetre size is relatively small, and that of millimetre or smaller size is relatively high, the impact of space debris of 0.1 mm size and above on the double satellite formation for gravity field exploration is investigated and analysed.

Two independent collisions obey an exponential distribution, then the probability density function of time is^9^

20 $$f\left( \lambda \right) = \lambda e^{ - \lambda t} .$$

**Figure 3:**
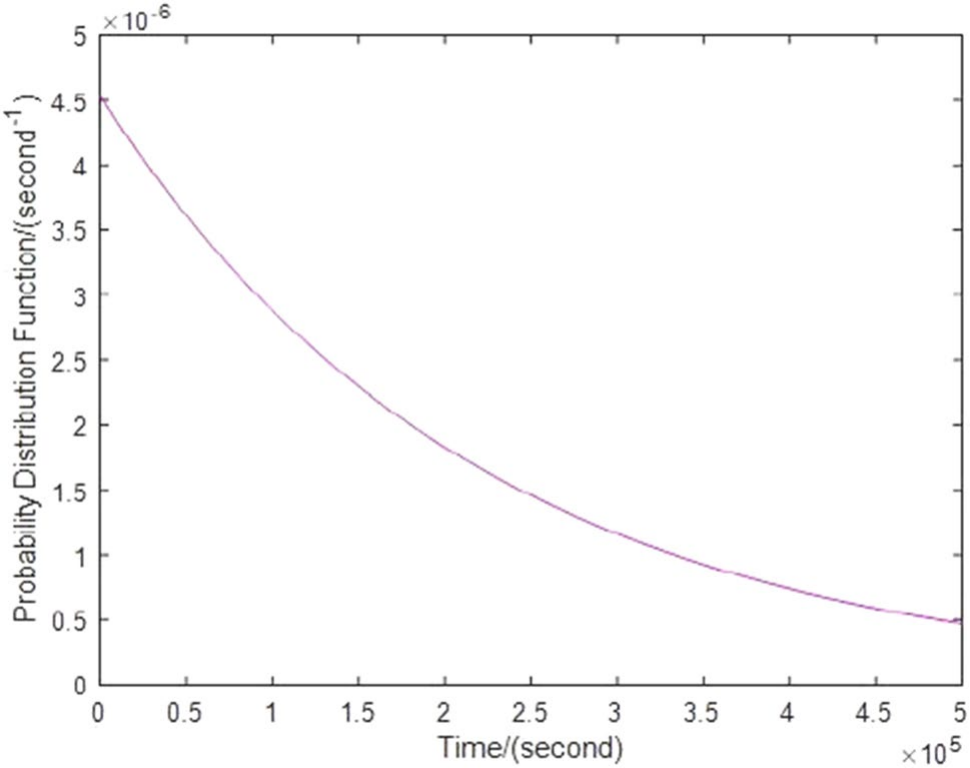
Probability density functions of time between independent collisions.

The number of space debris with size greater than or equal to 0.1 mm colliding the formation per unit time is $\lambda = 4.5314 \times 10^{ - 6} \,{\text{s}}^{ - 1}$.Therefore the probability density function of two independent collision times can be obtained from Eq. (20), as shown in Fig. 3.

The space debris impact the formation is a random event, obeying Poisson distribution, then the probability of double satellite formation being impacted $N$ times is

21 $$P\left( N \right) = \frac{{\lambda^{N} }}{N!}e^{ - \lambda }$$

If the probability density function $f_{X} \left( x \right)$ is a continuous function, then the probability of random variable $X$ in interval $a$ and $b$ is

22 $$P\left[ {a \le X \le b} \right] = \int_{a}^{b} {f_{X} \left( x \right)dx}$$

If the size of space debris (or its speed relative to the satellite) is within the interval $\left[ {a,b} \right]$, the probability of collision with the formation in this interval satisfies Eq. (22), and the angle function of impact is

23 $$P\left[ {a \le \theta \le b,\mathbf{c} \le \phi \le d} \right] = \int_{a}^{b} {\int_{c}^{d} {f_{\theta ,\phi } \left( {x,y} \right)dydx} } ,$$

where, $f_{\theta ,\phi } \left( {x,y} \right)$ represents the probability density function of altitude and azimuth angle, the range of altitude angle is $\left[ { - 90^{ \circ } ,90^{ \circ } } \right]$, and the range of azimuth angle is $\left[ { - 180^{ \circ } ,180^{ \circ } } \right]$.

Before and after the double satellite formation is impacted by space debris, the momentum of the system composed of the debris and the formation is conserved. Because the actual motion of the satellite after being hit by debris is more complex—both translational and rotational motion. For the convenience of the study, only the translational motion of the satellite is studied when the impact passes through the satellite center of mass. Only the rotation of the satellite is studied when it does not pass through the center of mass.

When the extension of the debris velocity passes the satellite center of mass, the momentum lost by the debris is equal to the increased momentum of the satellite. However, when the extension of the debris velocity does not pass through the satellite center of mass, the momentum lost by the debris is converted into the increased angular momentum of the satellite.

Assuming that at time $\tau$, the i-th space debris hits the satellite formation for a sustained impact time of $\varepsilon$, and that the change in linear momentum of the satellite during that time is ${\mathbf{P}}_{i}$ and the change in angular momentum is ${\mathbf{H}}_{i}$, the increase in force or moment of the satellite upon impact is

24 $$\left\{ {\begin{array}{*{20}l} {{\mathbf{F}}_{impact} \left( t \right) = \frac{{{\mathbf{P}}_{i} }}{\varepsilon }\left[ {\delta \left( {t - \tau } \right) - \delta \left( {t - \tau - \varepsilon } \right)} \right]\;\,\,} \\ {{\mathbf{M}}_{impact} \left( t \right) = \frac{{{\mathbf{H}}_{i} }}{\varepsilon }\left[ {\delta \left( {t - \tau } \right) - \delta \left( {t - \tau - \varepsilon } \right)} \right],} \\ \end{array} } \right.$$

where $\delta \left( t \right)$ is the unit step function. The relationship between angular momentum and momentum is ${\mathbf{H}} = {\mathbf{r}} \times {\mathbf{P}}$.

When the satellite is hit by debris at high speed, the direction of debris momentum does not pass through the satellite center of mass, the moment ${\mathbf{M}}_{impact}$ is obtained immediately after the satellite is hit, and the attitude and angular velocity of the satellite will change accordingly. When the direction of debris momentum passes through the satellite center of mass, the force ${\mathbf{F}}_{impact}$ will be obtained immediately after the satellite is hit, and the position and velocity of the impacted satellite will change accordingly.

This paper mainly studies the attitude stability analysis of formation after being impacted by debris. The angular velocity of the satellite after impact is

25 $${{\varvec{\upomega}}}_{A} = {{\varvec{\upomega}}}_{B} + {\text{d}} {{\varvec{\upomega}}},$$

where, ${{\varvec{\upomega}}}_{A}$ is the angular velocity of the satellite after impact, ${{\varvec{\upomega}}}_{B}$ is the angular velocity of the satellite before impact, and ${\text{d}}{{\varvec{\upomega}}}$ is the increased angular velocity of the satellite after impact.

Since the angular momentum transferred to the satellite is instantaneous when the space debris collides with the satellite. When the momentum of the debris after the collision with the satellite is all converted into the increased angular momentum of the satellite,

26 $${\mathbf{r}} \times {\mathbf{p}} = {\mathbf{J}} \cdot {\text{d}}{{\varvec{\upomega}}},$$

where, ${\mathbf{r}}$ is the vector from the satellite center of mass to the impact point, ${\mathbf{p}}$ is the momentum of the debris, and ${\mathbf{J}}$ is the rotational inertia of the satellite.

From Eqs. (25) and (26), the angular velocity of the spacecraft after impact is

27 $${{\varvec{\upomega}}}_{A} = {{\varvec{\upomega}}}_{B} + {\mathbf{J}}^{ - 1} \left( {{\mathbf{r}} \times {\mathbf{p}}} \right).$$

Assuming that the satellite formation is impacted by the $i$-th space debris whose the mass is $m_{i}$, the velocity is ${\mathbf{v}}_{i}$, the height angle is $\theta_{i}$, and an azimuth angle is $\phi_{i}$, and the linear momentum of the space debris is $p_{i}$ in the orbital frame can be expressed as

28 $${\mathbf{p}}_{i} = \left[ {\begin{array}{*{20}l} {p_{x} } \\ {p_{y} } \\ {p_{z} } \\ \end{array} } \right] = \left[ {\begin{array}{*{20}l} { - m_{i} v_{i} \cos \theta_{i} \cos \phi_{i} } \\ { - m_{i} v_{i} \cos \theta_{i} } \\ {m_{i} v_{i} \sin \theta_{i} } \\ \end{array} } \right].$$

Space debris has various shapes and types. For the convenience of research, this paper assumes that the debris is a sphere with a density of $\rho$, and the mass of the debris is

29 $$m_{i} = \frac{4}{3}\pi \left( {\frac{{l_{i} }}{2}} \right)^{3} \rho ,$$

where, $l_{i}$ is the size of the debris, subject to software ORDEM2000.

The impact of space debris on satellite is an inelastic collision process, in which momentum is conserved but energy is lost. Due to the complexity of the high-speed impact of space debris on satellites, this paper assumes that the impact of space debris on satellites is an inelastic collision process, in which momentum is conserved but energy is greatly lost.

The change of momentum after the satellite is impacted by debris is

30 $${\mathbf{P}}_{i} = {\mathbf{p}}_{i} .$$

In order to accurately calculate the angular momentum of the satellite after being impacted, it is necessary to know the vector ${\mathbf{r}}$. However, it is complicated to accurately calculate its specific expression. In this paper, it is assumed that the impact position is subject to uniform distribution in the impact plane, then the height angle and azimuth angle of the $i$-th space debris impact in plane $A_{j}$ are $\theta_{ji}$ and $\phi_{ji}$ respectively.

The probability of the $j \in \left\{ {1, \cdots ,6} \right\}$ plane of the satellite being impacted is

31 $$P_{j} = \frac{{A_{j} \cos \psi_{j} }}{{\sum\nolimits_{j = 1}^{6} {A_{j} \cos \psi_{j} } }},$$

where, $A_{j}$ is the area of the $j$-th plane, and $\psi_{j}$ is the angle between the momentum direction of the debris and the normal in the $j$-th plane before impact.

The expression of vector ${\mathbf{r}}$ in body frame is

32 $${\mathbf{r}}_{ji} = \left[ {\begin{array}{*{20}l} {x_{ji} } & {y_{ji} } & {z_{ji} } \\ \end{array} } \right]^{T} .$$

The two coordinates in $\left( {x_{ji} ,y_{ji} ,z_{ji} } \right)$ are uniformly distributed, and the other coordinate can be easily determined according to impact surface $A_{j}$.

In the process of high-speed collision, the momentum reduced by space debris is transferred to the satellite. At the same time, the sputter generated by debris collision will be ejected against the direction of the formation operation, taking away a small part of momentum. The change in the momentum of the system occurs as follows

33 $$\Delta {\mathbf{P}}_{i} = \frac{{{\mathbf{P}}_{i} \left( {t - \tau } \right)}}{1 - \xi } - {\mathbf{P}}_{i} \left( {t - \tau - \varepsilon } \right),$$

where, $\xi$ is the momentum enhancement factor, indicating the impact of the sputter on the transferred momentum after the satellite is impacted by space debris.

## Simulation and discussion

### Motion control simulation without debris impact in scientific mode

**Figure 4:**
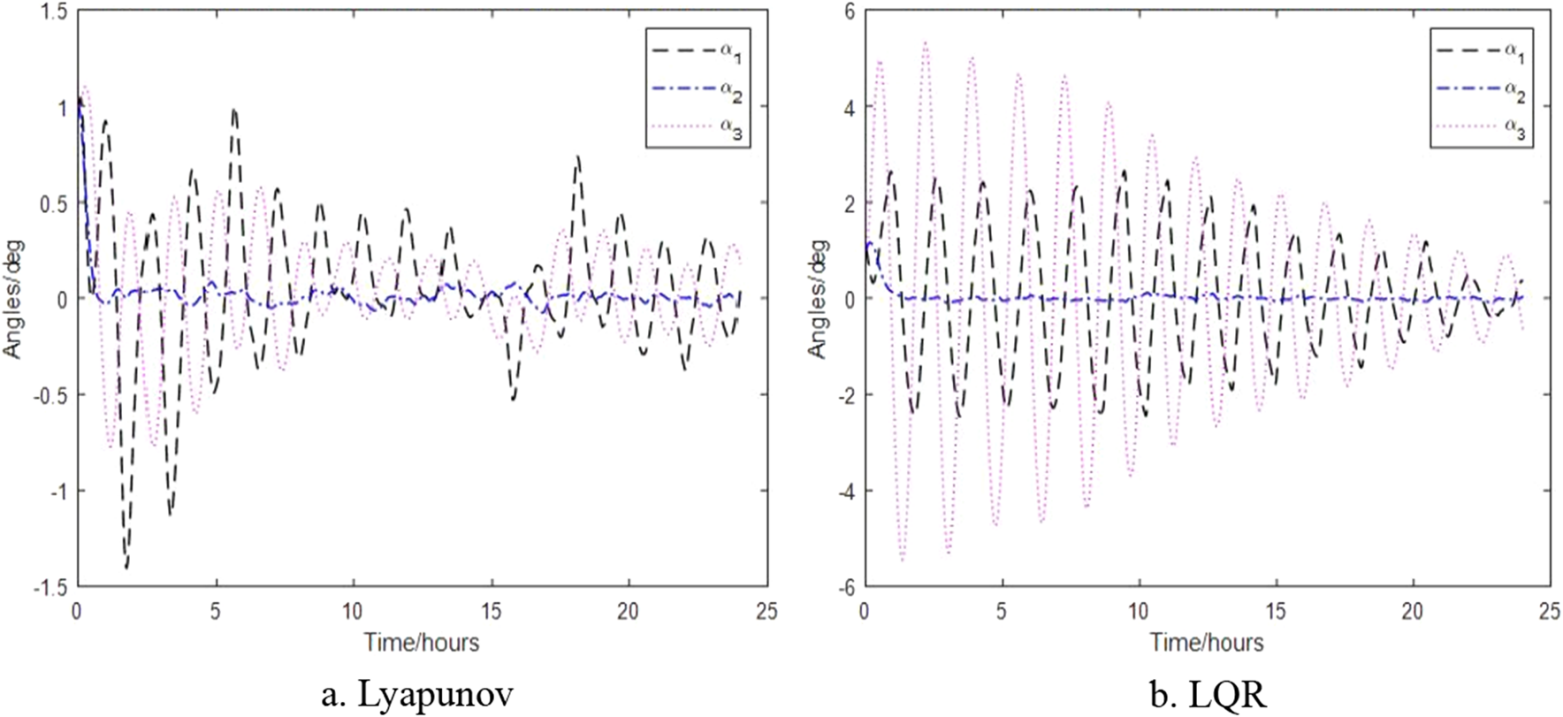
Attitude without thruster control.

**Figure 5:**
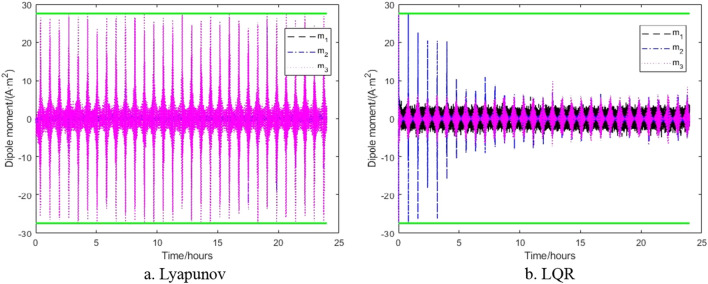
Magnetic dipole vector without thruster.

In order to verify the attitude controller developed in this paper, the parameters in Tables 1 and 2 were selected for simulation testing. The control parameters of the control algorithm based on Lyapunov's design are $${\mathbf{k}}_{\alpha } = 10^{ - 4} \times diag(3.8710,9.6774, - 1.6129)N\,m$$,$${\mathbf{k}}_{\omega } = diag(0.3387,1.01,0.1065)$$$$N\,m\,s$$ and based on LQR are $$Q = {\text{diag}}(1,1,1,1,15,10)$$, $$R = 10^{4} \times diag(10^{4} ,1,10^{3} ,1,1,1)$$ when the satellite is subjected to external torques of disturbance. The results of pure magnetic control are shown in Figs. 4 and 5.

**Table 1:** Initial values of spacecraft attitude and orbit.

Parameter	Symbol	Numerical Value	Unit
Tensor of inertia	$J_{1} ,J_{2} ,J_{3}$	110.4,580.5,649.5	kg m^2^
Tensor of inertia error	$$\left[ {\begin{array}{*{20}l} {\delta J_{11} } & {\delta J_{12} } & {\delta J_{13} } \\ {\delta J_{21} } & {\delta J_{22} } & {\delta J_{23} } \\ {\delta J_{31} } & {\delta J_{32} } & {\delta J_{33} } \\ \end{array} } \right]$$	$$\left[ {\begin{array}{*{20}l} 7 & {0.02} & {0.05} \\ {0.02} & {5.79} & {0.09} \\ {0.05} & {0.09} & 5 \\ \end{array} } \right]$$	kg m^2^
Sensitizer noise	$\delta \alpha$	diag(5^2^, 5^2^, 40^2^)	arcses^2^
Fiber optic gyro noise	$\delta \dot{\alpha }$	diag(5^2^, 5^2^, 5^2^)	(arcses/s)^2^
Orbit radius	$R$	6821	km
Orbital period	$T$	94	min
Double satellite distance	$\Delta r$	100	km
Orbit inclination	$i$	89	deg
Right ascension of ascending node	$\Omega$	78	deg
Geomagnetic inclination	$\gamma_{m}$	11.44	deg
Earth angular velocity	$\omega_{e}$	7.292 × ${10}^{-5}$	rad/s
Outline dimension	a/b/h/l	0.9/1.8/0.78/3.35	m

**Table 2:** Attitude control accuracy and maximum thrust torque.

	Control accuracy	Maximum torque
$\alpha_{1}$	0.14°	$1.5 \times 10^{ - 3} \;{\text{N}}\,{\text{m}}$
$\alpha_{2}$	0.014°	$1.5 \times 10^{ - 3} \,{\text{N}}\,{\text{m}}$
$\alpha_{3}$	0.014°	$1.5 \times 10^{ - 3} \,{\text{N}}\,{\text{m}}$

Figure 4 shows that the control algorithm designed by Lyapunov has higher control accuracy, and the control accuracy is within ± 1.5°, when the actuator is only the magnetorquer and all initial values are 1°, while the control algorithm designed by LQR is accurate within ± 6°. Both control algorithms show that the pitch axes have the highest control accuracy, as that there is always a magnetic torque control effect on this axis. Figure 5a shows that the magnetic dipole vector transitions frequently between saturated states ± 27.5 ${\text{A}}\,{\text{m}}^{2}$, while Fig. 5b shows that the magnetic dipole vector can initially reaches saturation, but later it is below ± 10 ${\text{A}}\,{\text{m}}^{2}$, indicating that the Lyapunov-based control algorithm is more effective.

**Figure 6:**
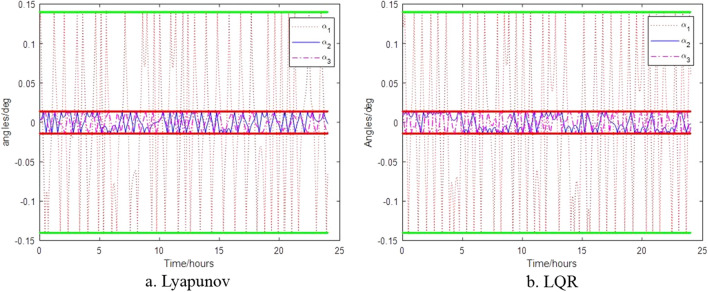
Control of satellite attitude with thruster action.

**Figure 7:**
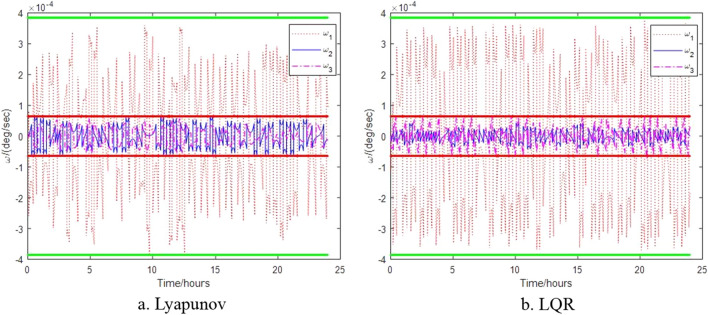
Control of angular velocity with thruster action.

**Figure 8:**
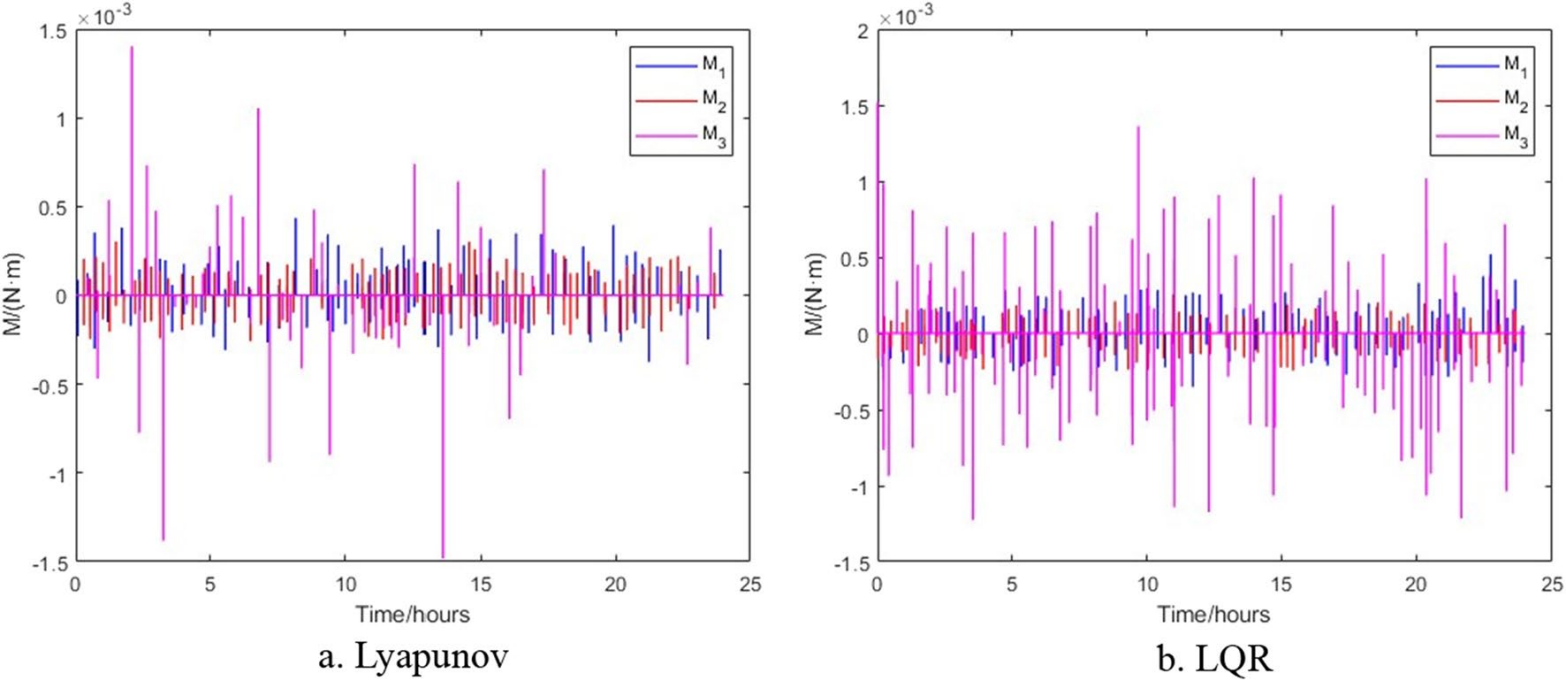
Thrust torque generated by thrusters.

The control results of the control algorithms based on Lyapunov and LQR when the thruster provides thrust torque are shown in Figs. 6, 7 and 8. Figure 6 shows the relative attitude control of the satellite achieved by the two control algorithms, and three axes can meet the attitude control accuracy. Figure 7 shows the relative angular velocity control of the satellite implemented by the two control algorithms, three axes also satisfy the angular velocity control requirement. Figure 8 shows the thrust torque required by the two control algorithms. Obviously, the torque required based on Lyapunov control algorithm is small, and the switching firing frequency is also low. Over a 24-h period, the total firing frequency of Lyapunov are 26 less than that of LQR, which is similar to the results of literature^17^.

### Monte Carlo simulation and critical momentum statistics

There are three changes to the control system after the double satellite formation being impacted by space debris. Firstly, the accuracy of the control does not vary significantly because the control system designed is somewhat robust. Secondly, the control accuracy exceeds the maximum allowed value, causing the system to exit the science mode. Thirdly, it causes the control system to diverge and the task to fail. Scientific mode means that the spacecraft is in the normal operation stage. Exit from scientific mode means that the spacecraft can not work normally, but may return to normal operation after relevant control.

**Figure 9:**
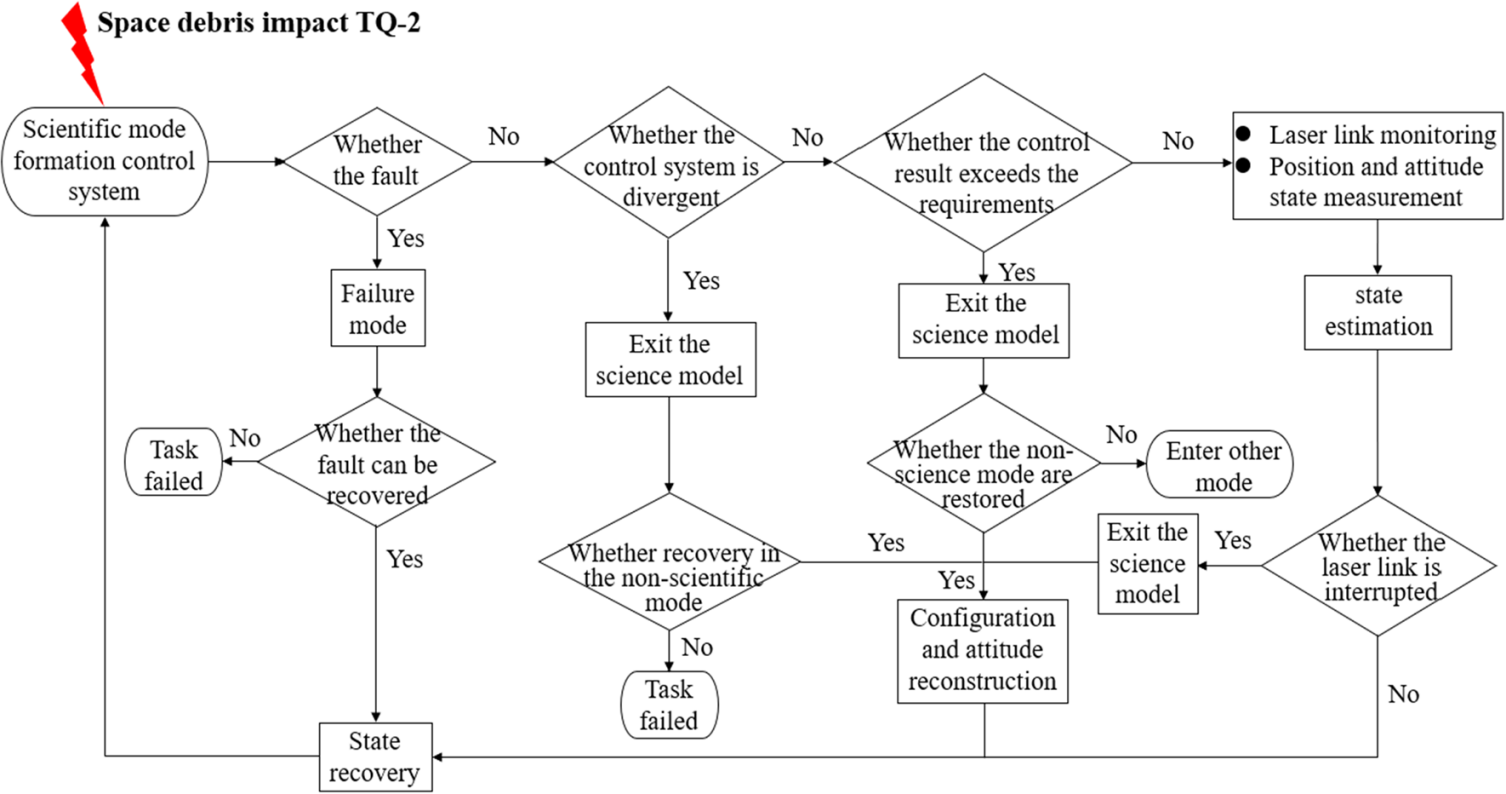
Logic diagram of the control system after impact by debris.

The logical relationship of the change in the control system of the double satellite formation after a high-speed impact of space debris is shown in Fig. 9.

**Figure 10:**
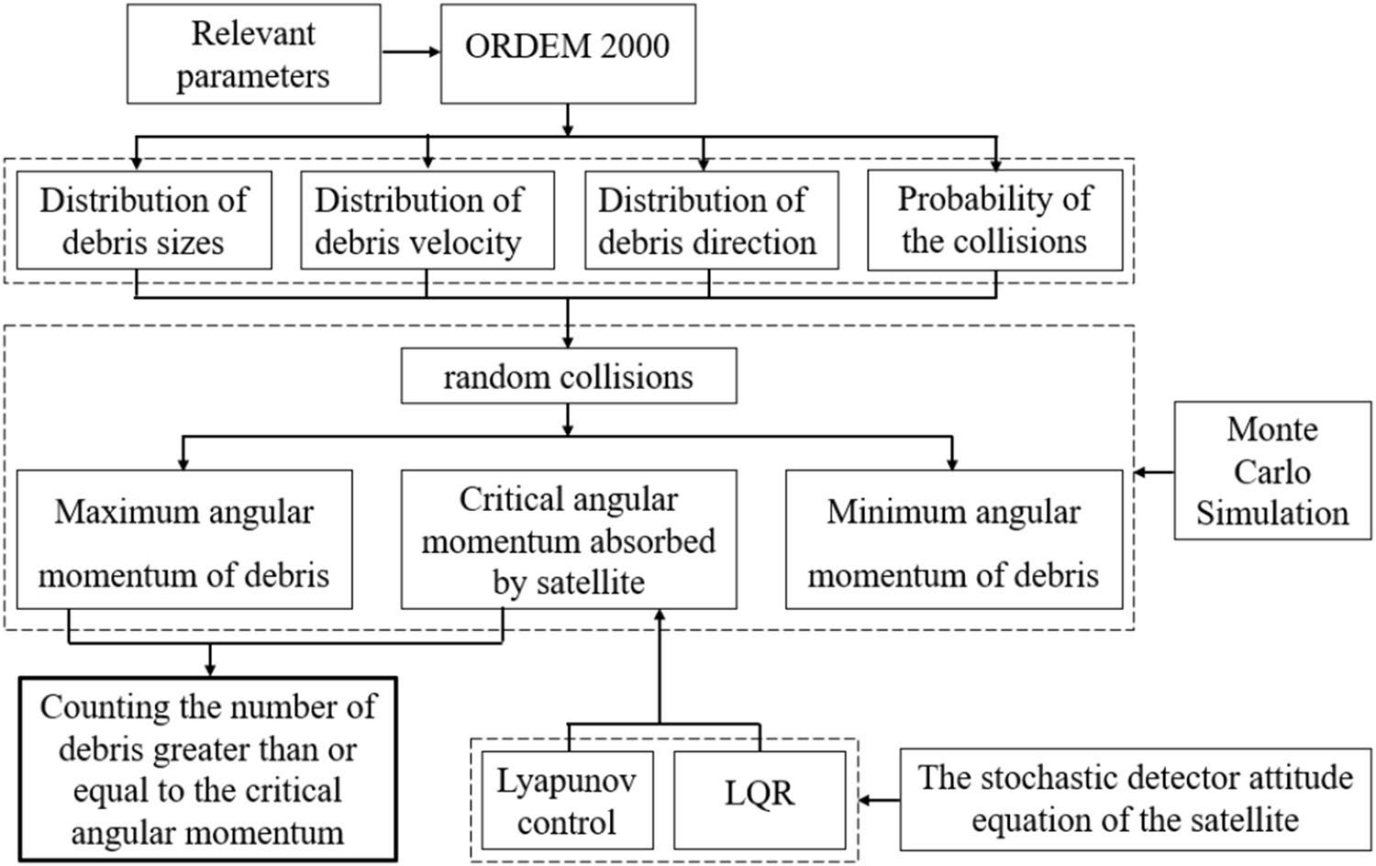
Relationship between attitude control and Monte Carlo simulation.

The critical momentum referred to is the momentum that the impact of space debris happens to cause the satellite to exit the science mode. As soon as one degree of freedom exits the science mode during the collision, we consider that the momentum gained by this impact on the satellite exceeds the critical momentum. The relationship between the Monte Carlo simulation and the control system is shown in Fig. 10.

The density of space debris is taken as $\rho = 2.8\,{{\text{g}} \mathord{\left/ {\vphantom {{\text{g}} {{\text{cm}}^{3} }}} \right. \kern-0pt} {{\text{cm}}^{3} }}$, assuming a collision duration of $\varepsilon = 0.1\,{\text{s}}$, which occurs at $\tau = 18,000\,{\text{s}}$. The area of each surface of the satellite is shown in Table 3.

**Table 3:** Area of each surface of the satellite.

Surface	Area
Upper surface	3.105 ${\text{m}}^{2}$
Lower surface	6.03 ${\text{m}}^{2}$
Front surface	1.053 ${\text{m}}^{2}$
Rear surface	1.053 ${\text{m}}^{2}$
Left surface	3.105 ${\text{m}}^{2}$
Right surface	3.105 ${\text{m}}^{2}$

**Figure 11:**
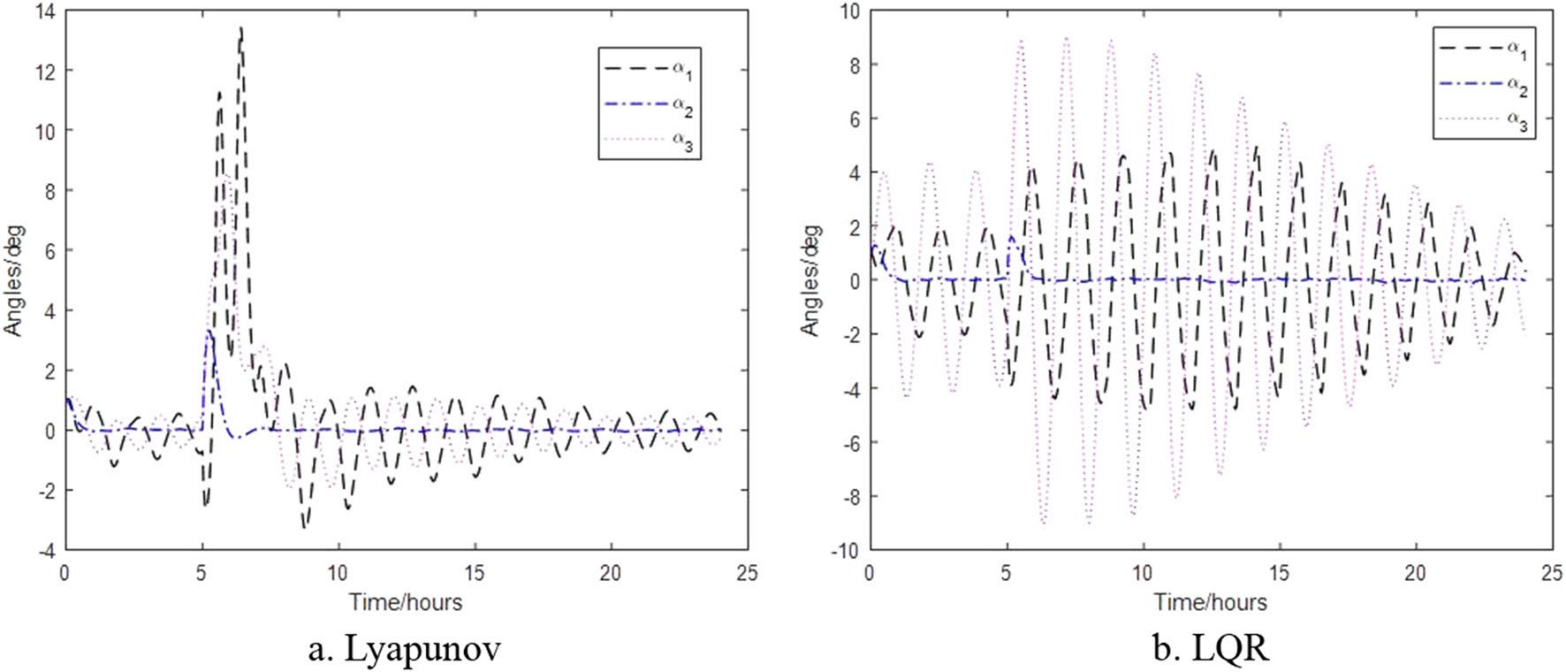
Attitude without thruster control after space debris impact.

The thrustless attitude control of the satellite is shown in Fig. 11 when the satellite is hit by debris with an increased angular momentum of $L_{1} = - 1.8016 \times 10^{ - 4} \,{{{\text{kg}}\,{\text{m}}^{2} } \mathord{\left/ {\vphantom {{{\text{kg}}\,{\text{m}}^{2} } {\text{s}}}} \right. \kern-0pt} {\text{s}}}$, $L_{2} = 1.6135 \times 10^{ - 4} \,{{{\text{kg}}\,{\text{m}}^{2} } \mathord{\left/ {\vphantom {{{\text{kg}}\,{\text{m}}^{2} } {\text{s}}}} \right. \kern-0pt} {\text{s}}}$ and $L_{3} = 0.8253 \times 10^{ - 4} \,{{{\text{kg}}\,{\text{m}}^{2} } \mathord{\left/ {\vphantom {{{\text{kg}}\,{\text{m}}^{2} } {\text{s}}}} \right. \kern-0pt} {\text{s}}}$ respectively. Figure 11a shows that the Lyapunov-based control algorithm has a large attitude change within a short period of time after impact, but quick plateaus. While Fig. 11b shows that the LQR-based control algorithm takes a long time to plateau after impact, which further illustrates the control law of high control accuracy and low robustness.

**Figure 12:**
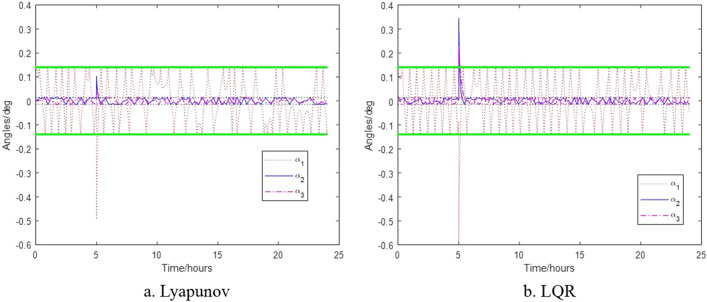
Satellite attitude after space debris impact.

**Figure 13:**
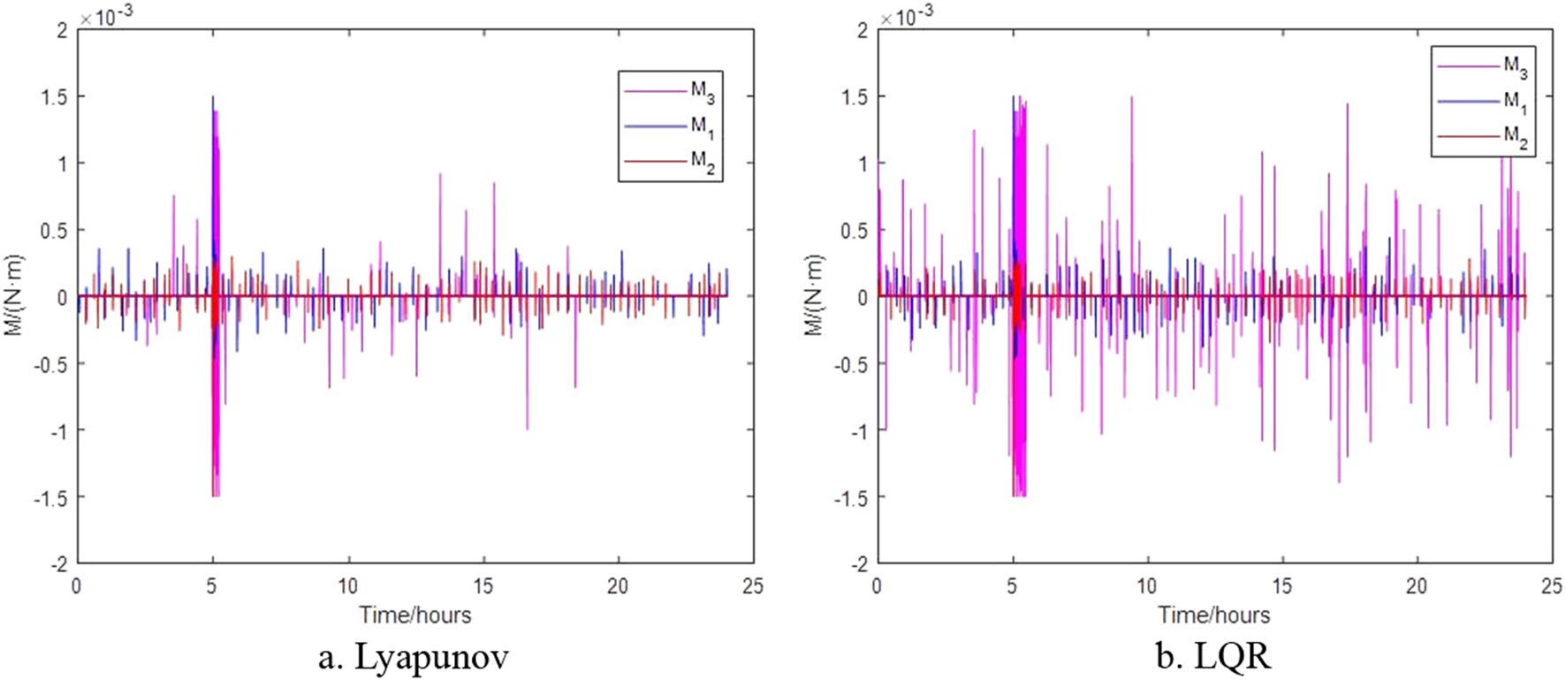
Thrust torque after space debris impact.

The change in attitude and thrust moment of the satellite when it acquires angular momentum of $L_{1} = - 1.7921 \times 10^{ - 2} \,{{{\text{kg}}\,{\text{m}}^{2} } \mathord{\left/ {\vphantom {{{\text{kg}}\,{\text{m}}^{2} } {\text{s}}}} \right. \kern-0pt} {\text{s}}}$, $L_{2} = 1.1543 \times 10^{ - 2} {{{\text{kg}}\,{\text{m}}^{2} } \mathord{\left/ {\vphantom {{{\text{kg}}\,{\text{m}}^{2} } {\text{s}}}} \right. \kern-0pt} {\text{s}}}$ and $L_{3} = 0.7861 \times 10^{ - 2} \,{{{\text{kg}}\,{\text{m}}^{2} } \mathord{\left/ {\vphantom {{{\text{kg}}\,{\text{m}}^{2} } {\text{s}}}} \right. \kern-0pt} {\text{s}}}$ after impact are shown in Figs. 12 and 13 respectively. Figure 12 shows that when space debris with the same momentum hits the satellite formation, the attitude will exceed the requirements under the different control. Figure 13 shows that the control algorithm based on Lyapunov control can return to normal thrust conditions within 25 min, while the control algorithm based on LQR takes 48 min for the thrusters to return to normal thrust conditions under the impact of space debris of same momentum.

**Figure 14:**
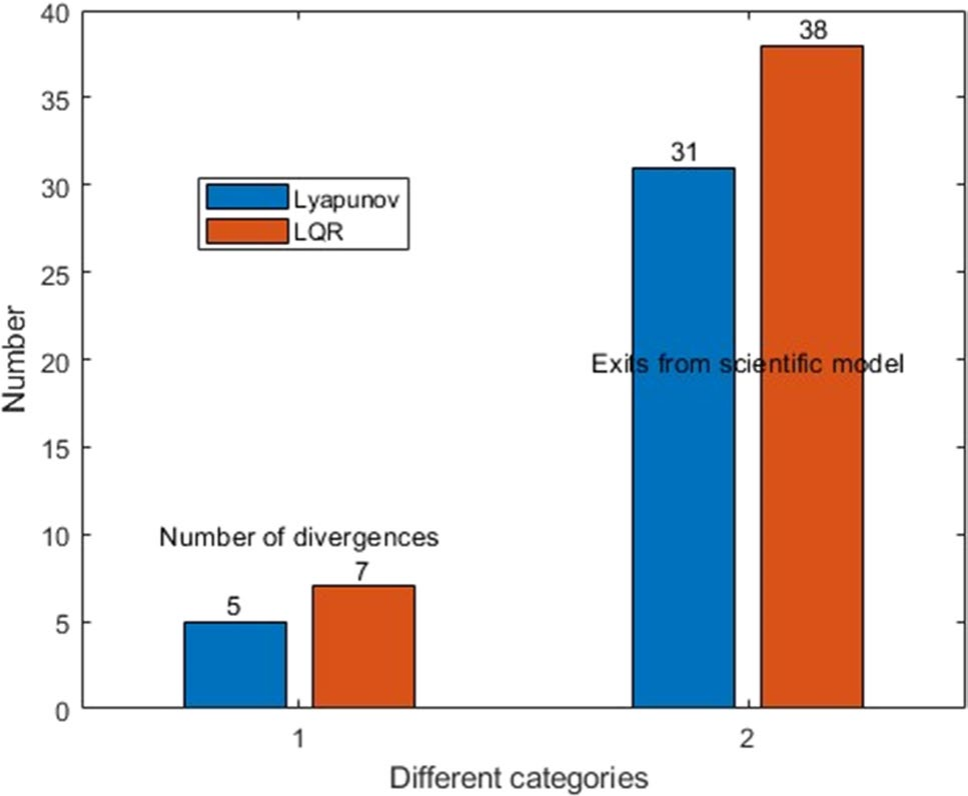
The number of times the impact that causes the formation to exit the scientific mode and diverge under different control algorithms.

**Figure 15:**
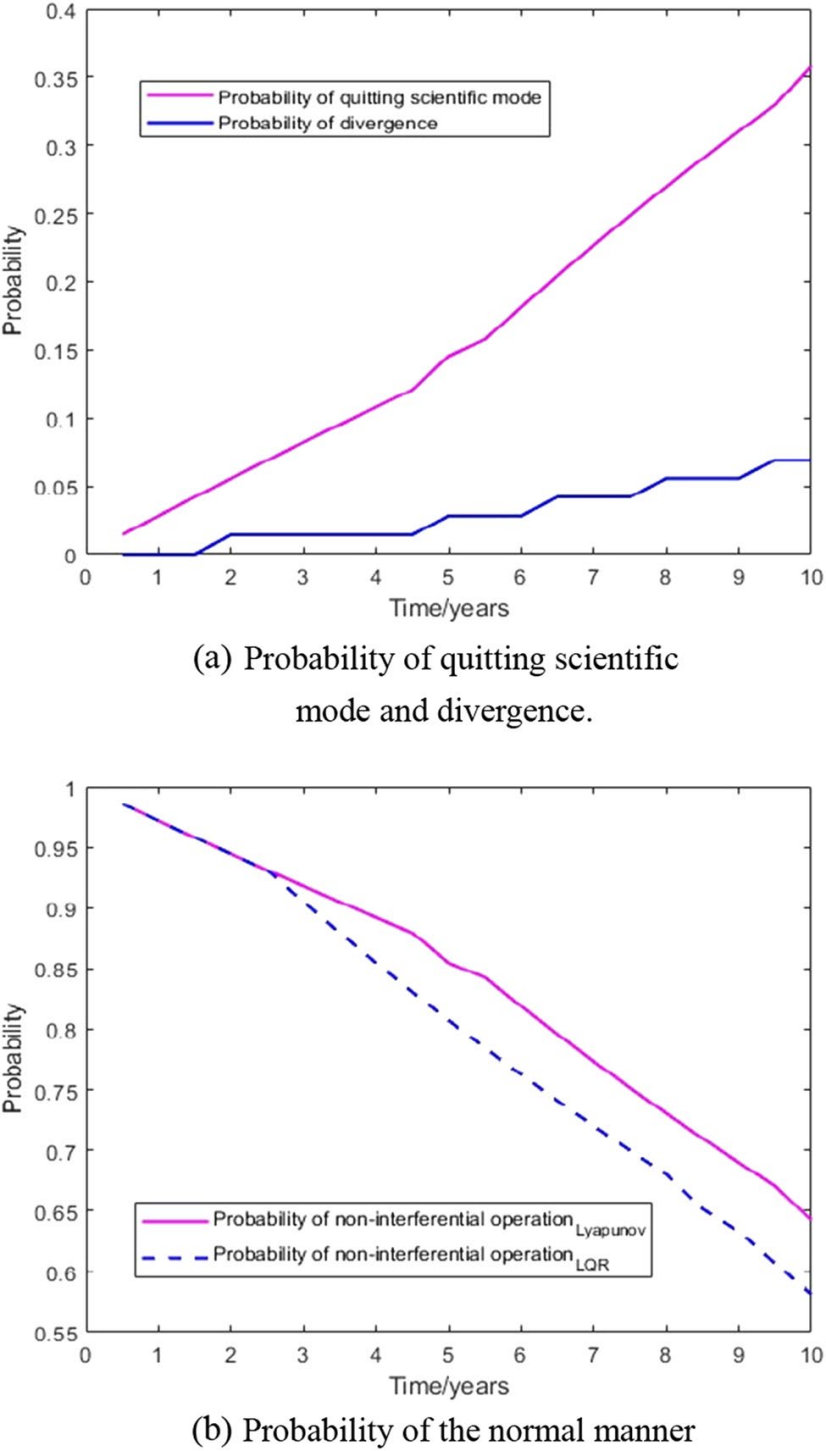
Probability of impact exiting science mode, divergence and normal manner of double satellite formation relative to attitude.

The process of Monte Carlo simulation and statistics is shown in Fig. 10. In this paper, 100,000 data satisfying the size distribution, velocity distribution and angle distribution of space debris software ORDEM2000 are selected. These data and the double satellite formation at the average orbital altitude randomly collide, and the probability density of two collision times conforms to the rule of Fig. 3. The results are as shown in Fig. 14 when Lyapunov control is adopted, the number of impacts greater than or equal to the critical angular momentum is 31, of which 5 times cause the control system to diverge. When LQR control is adopted, the number of impacts greater than or equal to the critical angular momentum is 38, of which 7 times cause the control system to diverge. Figure 15 shows that the probability of the double satellite formation exiting the science mode and divergence due to at least one impact is as shown in Fig. 15a. Figure 15b shows that the Lyapunov-based control algorithm has higher stability after being hit. In the 10-year mission period, the formation for gravity field exploration has been impacted by 1428 space debris with size greater than or equal to 0.1 mm. For the two control algorithms, debris impact can cause the system to exit the science mode and cause the control system to diverge. There is a slight difference in the probability of normal manner, but the difference is small when using different control algorithms.

## Conclusion

In this paper, the stability of relative attitude of the double satellite formation for gravity field in space debris environment is studied. We established the dynamical equation of random collision of relative attitude, and adopted two control algorithms, Lyapunov control and LQR. Under the corresponding conditions, the space debris distribution function is established with the international space debris software, and the probability density function of the time of two independent collisions is obtained according to the corresponding conditions. The impact of debris on the attitude control system was simulated by Monte Carlo simulation with 100,000 data satisfying the model. The results show that during the 10-year mission period, using the control algorithm designed by Lyapunov control, 31 impacts caused the satellite to exit science mode, 5 impacts caused its control to diverge. Based on the LQR, 38 impacts caused the satellite to exit the science mode, and 7 impacts caused the control system to diverge. This shows that the probability of satellite being knocked over vary in a small range due to different control algorithms, the two algorithms that meet the attitude control accuracy will both exit the science mode and unstability in the space debris environment. The control system still has the risk of interrupting the scientific detection mode. It is necessary to consider the satellite operation and maintenance technology and further study the countermeasures.

## Data Availability

The datasets used and/or analysed during the current study available from the corresponding author on reasonable request.
